# Tofacitinib and metformin reduce the dermal thickness and fibrosis in mouse model of systemic sclerosis

**DOI:** 10.1038/s41598-022-06581-1

**Published:** 2022-02-15

**Authors:** Ahmet Karatas, Burak Oz, Cigdem Celik, Zeynel Abidin Akar, Ramazan Fazil Akkoc, Ebru Onalan Etem, Adile Ferda Dagli, Suleyman Serdar Koca

**Affiliations:** 1 Department of Rheumatology, Firat University School of Medicine, Elazig, Turkey; 2 Department of Rheumatology, Fethi Sekin City Hospital, Elazig, Turkey; 3 Department of Internal Medicine, Gemlik State Hospital, Bursa, Turkey; 4 Department of Rheumatology, Gazi Yasargil Egitim ve Arastirma Hastanesi, Diyarbakir, Turkey; 5 Department of Anatomy, Firat University School of Medicine, Elazig, Turkey; 6 Department of Medical Biology, Firat University School of Medicine, Elazig, Turkey; 7 Department of Pathology, Firat University School of Medicine, Elazig, Turkey

**Keywords:** Connective tissue diseases, Systemic sclerosis, Medical research, Rheumatology

## Abstract

Janus kinase (JAK)-signal transducer and activator of transcription (STAT) pathway is important in the process of inflammation and fibrosis. The adenosine 5′-monophosphate-activated protein kinase (AMPK) enzyme can affect JAK/STAT pathway. Tofacitinib is a pan-JAK inhibitör. Metformin activates AMPK enzyme. We aimed to investigate the therapeutic efficacy of tofacitinib and metformin on IL-17 and TGF-β cytokines, skin fibrosis and inflammation in mouse model of systemic sclerosis (SSc). 40 Balb/c female mice were divided into 4 groups: (control, sham (BLM), tofacitinib and metformin). The mice in the tofacitinib group received oral tofacitinib (20 mg/kg/daily) and mice in the metformin group received oral metformin (50 mg/kg/day) for 28 days. At the end of 4th week, all groups of mice were decapitated and tissue samples were taken for analysis. Histopathological analysis of skin tissue was performed, and mRNA expressions of collagen 3A, IL-17 and TGF-β were assessed by real-time PCR and ELISA. Repeated BLM injections had induced dermal fibrosis. Moreover, the tissue levels of collagen 3A, IL-17 and TGF-β were elevated in the BLM group. Tofacitinib and metformin mitigated dermal fibrosis. They reduced dermal thickness and tissue collagen 3A, IL-17 and TGF-β levels. Tofacitinib and metformin demonstrated anti-inflammatory and anti-fibrotic effects in the mouse model of SSc.

## Introduction

Systemic sclerosis (SSc) is a systemic, inflammatory disease that has a progressive course starting with microvascular injury and inflammation in tissues leading to fibrosis. Although it is still not fully elucidated, the complex relationship among T cells, B cells, monocyte/macrophage, and fibroblasts is emphasised in pathogenesis of SSc^1–3^.

Composed of Janus kinase (JAK) and signal transducer and activator of transcription (STAT) components, JAK/STAT is an important signalling pathway in cytokine signalling. JAKs activate STATs by phosphorylation. The STAT family acts a role in cell proliferation and differentiation. In recent years, it has been suggested that the JAK/STAT signalling pathway is important in the process of fibrosis^4,5^. The JAK/STAT signalling pathway is demonstrated to be active in SSc in fibroblasts. In addition, the JAK/STAT signalling pathway acts an important role in activating profibrotic macrophages^6^. The role of canonical (SMAD) and non-canonical (non-SMAD) pathways in the process of transforming growth factor beta (TGF-β) mediated fibrosis is known. Non-canonical pathway associated with fibrosis occurs through JAK/STAT, mitogen activated protein kinases (MAPKs) (ERK, p38 and JNK), phosphatidylinositol-3-kinase (PI3K) and Rho-like GTPases^7,8^.

JAKs are a family of kinases that include JAK1, JAK2, JAK3, and tyrosine kinase 2 (TYK2). Tofacitinib (TOFA) is a potent inhibitor of JAK1 and JAK3 and is the first-generation JAK inhibitor that inhibits JAK2 and TYK2 with less potency. TOFA is licenced in the treatment of rheumatoid arthritis, psoriatic arthritis, and ulcerative colitis. Through the inhibition of JAK1 and JAK3, TOFA reduces cytokine-mediated Th1, Th2 Th17 differentiation and production of pro-inflammatory mediators^9–11^. Metformin (MET) is an antidiabetic treatment agent that inhibits the mammalian target of rapamycin (mTOR) by activating the adenosine 5′-monophosphate activated protein kinase (AMPK) enzyme. AMPK enzyme can inhibit JAK and STAT phosphorylation directly or through different mechanisms, such as mTOR, tuberous sclerosis complex 2 (TSC2) and MAP kinase-1^12^. As a result, TOFA and MET can suppress the processes of inflammation and fibrosis by affecting T lymphocyte differentiation through the JAK/STAT pathway and TGF-β mediated non-canonical fibrosis pathway.

Considering the effects of JAK/STAT pathway in the pathogenesis of inflammation and fibrosis, our study aimed to determine the effects of TOFA and MET treatments that inhibit the JAK/STAT pathway on IL-17 and TGF-β cytokines, skin fibrosis and inflammation by creating an experimental scleroderma model. According to current literature data, there is no study evaluating the effectiveness of these two treatment agents together in the scleroderma model created with bleomycin (BLM).

## Material and methods

### Animals and experimental applications

40 Balb/c female mice those were aged 6 to 8 weeks olds and weighing 20 to 25 g, were taken from Fırat University Experimental Research Center (FUDAM). All experimental applications were carried out in accordance with ARRIVE guidelines. Mice housed under suitable conditions were separated into four groups as control group, sham group, tofacitinib group and metformin group. A designated area in the dorsal area of all the mice was shaved for subcutaneous injections. Phosphate-buffered saline (PBS) 100 μL/day was injected subcutaneously to the mice in the control group for 4 weeks, while 100 μg BLM (in 100 μL PBS) to the mice in the remaining 3 groups was administered subcutaneously daily for 4 weeks^13^.

The body weights of the mice were evaluated daily. The mice in the TOFA group were administered TOFA at a daily dose of 20 mg/kg, while metformin at a daily dose of 50 mg/kg was administered in the mice of MET group. TOFA and MET were administered by oral way, for 28 days^14,15^.

Mice in all groups were decapitated under anaesthesia via intraperitoneal administration of ketamine (75 mg/kg) plus xylazine (10 mg/kg) at the end of the fourth week. Subsequently, the skin tissues of the mice were rapidly harvested for histopathological evaluations, analyses of tissue reverse transcription (RT)-PCR and ELISA. Skin tissues for histopathological evaluations were fixed with appropriate fixatives and then embedded in paraffin blocks, after undergoing histological follow-up series. We used 100 mg fresh tissue from each of the 40 skin samples for ELISA analyses. Samples were homogenized in phosphate buffer as described earlier^16^. On the other hand, skin tissue samples harvested for analysis of tissue mRNA expressions were stored, until the day of analysis, at − 80 °C. Skin tissue mRNA expressions were analysed by real-time PCR method. Ethical approval was obtained from Fırat University Animal Experiments Local Ethics Committee for the study, animal experimentation guidelines were exactly followed.

### Histopathological evaluations

Sections were prepared from paraffin blocks of tissue samples. Stained sections with Hematoxylin–Eosin and Masson–Trichrome were determined by an expert pathologist under light microscope (Olympus BX-50) at 40×, 100×, 200× and 400× magnification to detect the inflammatory cell infiltration and the degrees of fibrosis. For dermal thickness (the length between the epidermo–dermal junction and the dermis–subcutaneous adipose tissue junction), the average of at least five different measurements in at least two different preparations at 100× magnification was taken in each subject.

### Determination of tissue mRNA levels

The mRNA expressions of IL-17A, collagen 3A and TGF-β were determined by RT-PCR method, using an appropriate RNA isolation kit, from the tissue homogenate collected for real-time polymerase chain reaction analysis.

Trizol (Invitrogen, Carlsbad, CA, USA) was performed for RNA isolation in skin samples. Qubit RNA Assay Kit For Use With The Qubit 2.0 Fluorometer (Invitrogen, Carlsbad, CA, USA) was applied for RNA measurements. The amount of RNA was measured as μg/mL. In order to equalise the RNA amounts for complementary DNA (cDNA) synthesis, the lowest RNA value read was determined as standard. A RNA pool was designed from each samples group for synthesis of cDNA. The High-Capacity cDNA Reverse Transcription Kit (Applied Biosystems, Foster City, CA, USA) was used for synthesis of cDNA. The cDNAs obtained by reverse transcription were amplified in the presence of sequence specific primers TGF-β1 (Mm01178820_m1, Applied Biosystems, Foster City, CA, USA), ınterleukin 17A (IL-17A) (Mm00506606_m1, Applied Biosystems, Foster City, CA, USA), and Collagen Type III Alpha 1 Chain (COL3A1) (Mm00802305_g1, Applied Biosystems, Foster City, CA, USA) using Tag Man Master Mix (Applied Biosystems, Foster City, CA, USA) in the ABI Prism 7500 Fast Real-Time PCR device (Applied Biosystems, Foster City, CA, USA). Temperature conditions were set to be 2 min at 50 °C, 10 min × 40 cycles at 95 °C, 15 s at 95 °C and 1 min at 60 °C. Real-Time PCR was tested three times. Glyceraldehyde 3-phosphate dehydrogenase (GAPDH) (Mm99999915_g1, Applied Biosystems, Foster City, CA, USA) was used as a control housekeeping gene. The levels of gene expressions were determined by the comparative Ct (ΔCt) method^17,18^.

### ELISA analyses of the tissues

Mouse Interleukin 17 (IL-17) (Catalog no: ELK1147) and Collagen Type III Alpha 1 (COL3a1) (Catalog no: ELK7408), kits were obtained from ELK Biotechnology (Wuhan, CHINA). ELISA measurements were carried out in line with the procedures specified in the catalogs of the kits. Skin samples were assayed using a plate reader (Multiskan FC, Thermo Fisher Scientific, Waltham, MA, USA). The measurement range (detection range) of the IL-17 ELISA kit was 7.82–500 pg/mL, minimum measurable level (sensitivity) was 3.2 pg/mL, while the detection range of the COL3a1 ELISA kit was 15.63 -1000 pg/mL, the minimum measurable level (sensitivity) was 10.511 pg/mL. Additionally, all the kits used here had an Intra-Assay CV value of < 8% and an Inter-Assay CV value of < 10%.

### Statistical analysis

Statistical analysis were done via the IBM-SPSS statistics programme. The significance of possible differences between groups was evaluated using Kruskal–Wallis and Post-Hoc Mann–Whitney U tests. A p value of < 0.05 was considered statistically significant.

## Results

### Tofacitinib and metformin treatments corrected inflammation, skin thickness and dermal fibrosis in the SSc mouse model

Compared to the control group, the number of myofibroblasts increased by 447% and lymphocyte count increased by 41% in the BLM-applied sham group (p = 0.001 and p = 0.024, respectively). On the other hand, the number of myofibroblasts and lymphocytes decreased by 71% and 25% in the TOFA group when compared with the BLM group (p < 0.001 and p = 0.061, respectively). In the MET group, when compared with the BLM group, the number of myofibroblasts decreased by 67%, while the lymphocyte count decreased by 3% (p < 0.001 and p = 0.799, respectively) (Table 1).

**Table 1:** Histopathological findings in the study groups.

	PBS (n = 10)	BLM (n = 10)	TOFA (n = 10)	MET (n = 10)	p
Dermal thickness (µm)	131.4 ± 18.6	326.6 ± 96.1***	212.2 ± 54.4*^,≠^	220 ± 20.7*^,≠≠^	< 0.001
Myofibroblast (n/HPF)	1.5 ± 1.1	8.2 ± 2.4**	2.4 ± 0.7^≠≠≠^	2.7 ± 0.7^≠≠≠^	< 0.001
Lymphocytes (n/HPF)	1.57 ± 0.3	2.22 ± 0.44**	1.66 ± 0.70^≠^	2.16 ± 0.4	0.036

*BLM* bleomycin, *MET* metformin, *PBS *phosphate-buffered saline, *TOFA tofacitinib*, *HPF* high-power field.

Data were expressed as mean ± standard deviation.

When compared with the PBS group: *p < 0.05, **p < 0.01, ***p < 0.001.

When compared with the BLM group: ^≠^p < 0.05, ^≠≠^p < 0.01, ^≠≠≠^p < 0.001.

**Figure 1:**
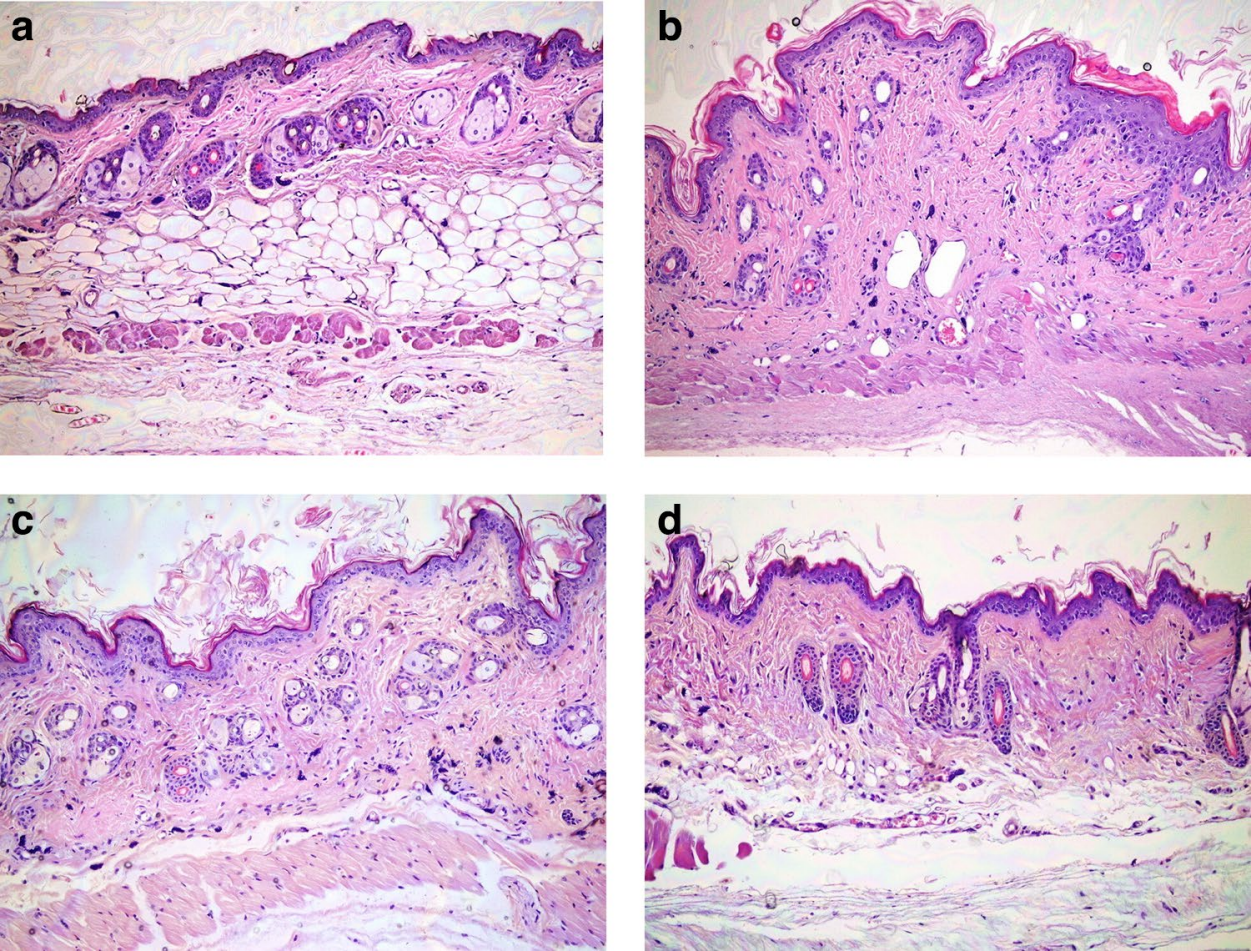
Histopathological studies from sections of skin in the study groups (hematoxylin and eosin staining, ×100). Normal histopathological view of mice immunised with PBS in control group (**a**). Increased dermal thickness, infiltration of inflammatory cell and fibrosis in the BLM-injected sham group (**b**). Tofacitinib (**c**) and metformin (**d**) applications decreased the dermal thickness, infiltrations of inflammatory cell and fibrosis in the BLM-injected mice.

**Figure 2:**
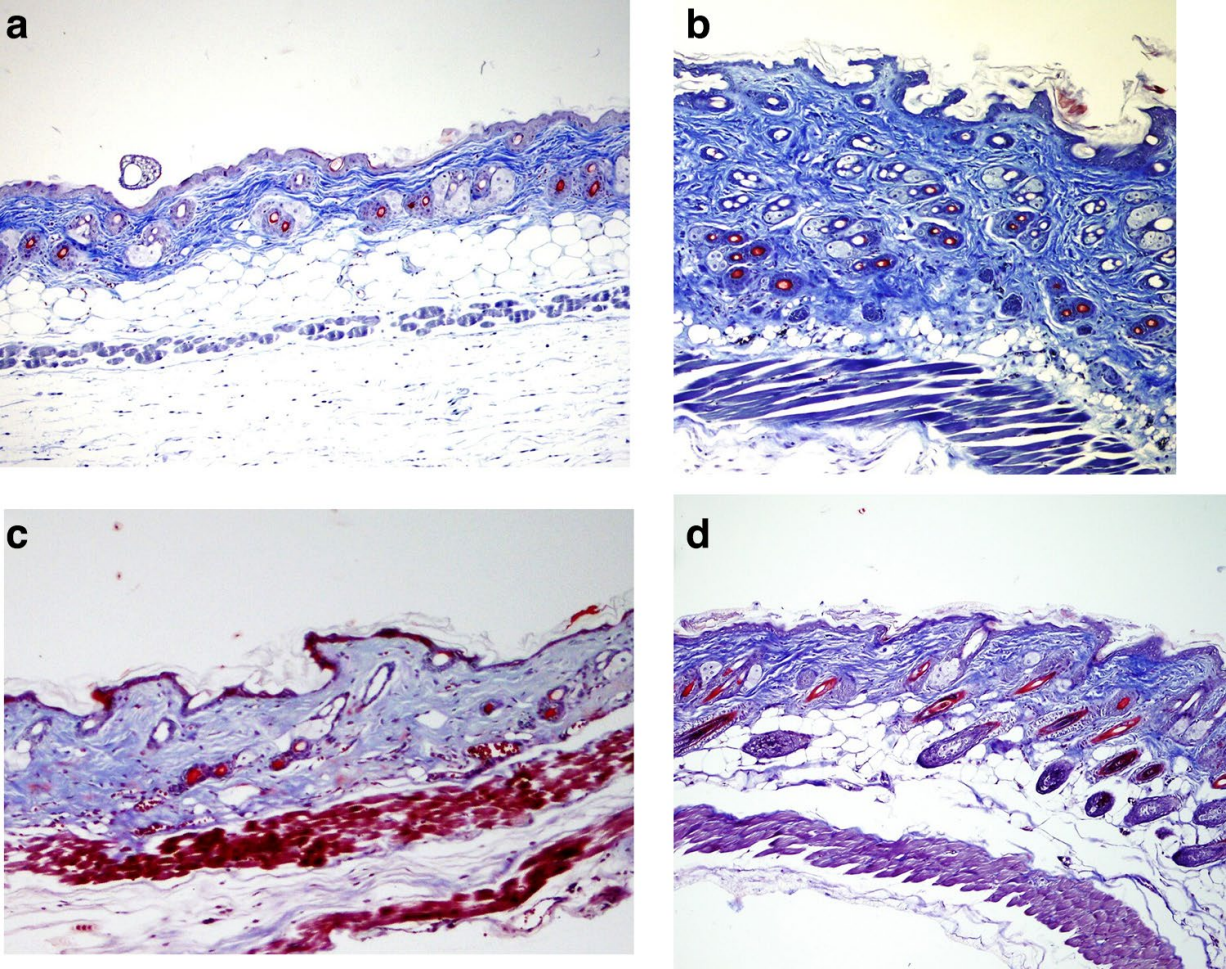
Dermal fibrosis in the study groups (Masson’s Trichrom staining, ×100). Normal histopathological view of mice immunized with PBS in control group (**a**). Increased dermal thickness, infiltration of inflammatory cell, fibrosis in the BLM-injected sham group (**b**). Tofacitinib (**c**) and metformin (**d**) applications decreased the dermal thickness, infiltrations of inflammatory cells, and fibrosis in the BLM injected mice.

Fibrosis was not observed in the histopathological evaluation of the skin tissue of mice in the first group that received PBS injection alone (Figs. 1a, 2a). In addition, BLM injections lead to increased inflammatory cell infiltration and dermal fibrosis, in the dermal and subcutaneous areas. Dermal fibrosis was more evident in the BLM-applied sham group (Figs. 1b, 2b). Dermal thickness increased by 149% in the BLM-applied sham group when compared to the control group (p = 0.001).

Dermal fibrosis was resolved with both TOFA and MET treatment compared to the BLM-applied sham group (Figs. 1c,d, 2c,d). The dermal thickness was reduced by 35% in the TOFA group (p = 0.024) and was reduced by 32% in the MET-applied group (p = 0.007), when compared with the BLM-applied sham group (Table 1).

### Tofacitinib and metformin treatments decreased IL-17, collagen 3a1 and TGF-βlevels in the skin

**Figure 3:**
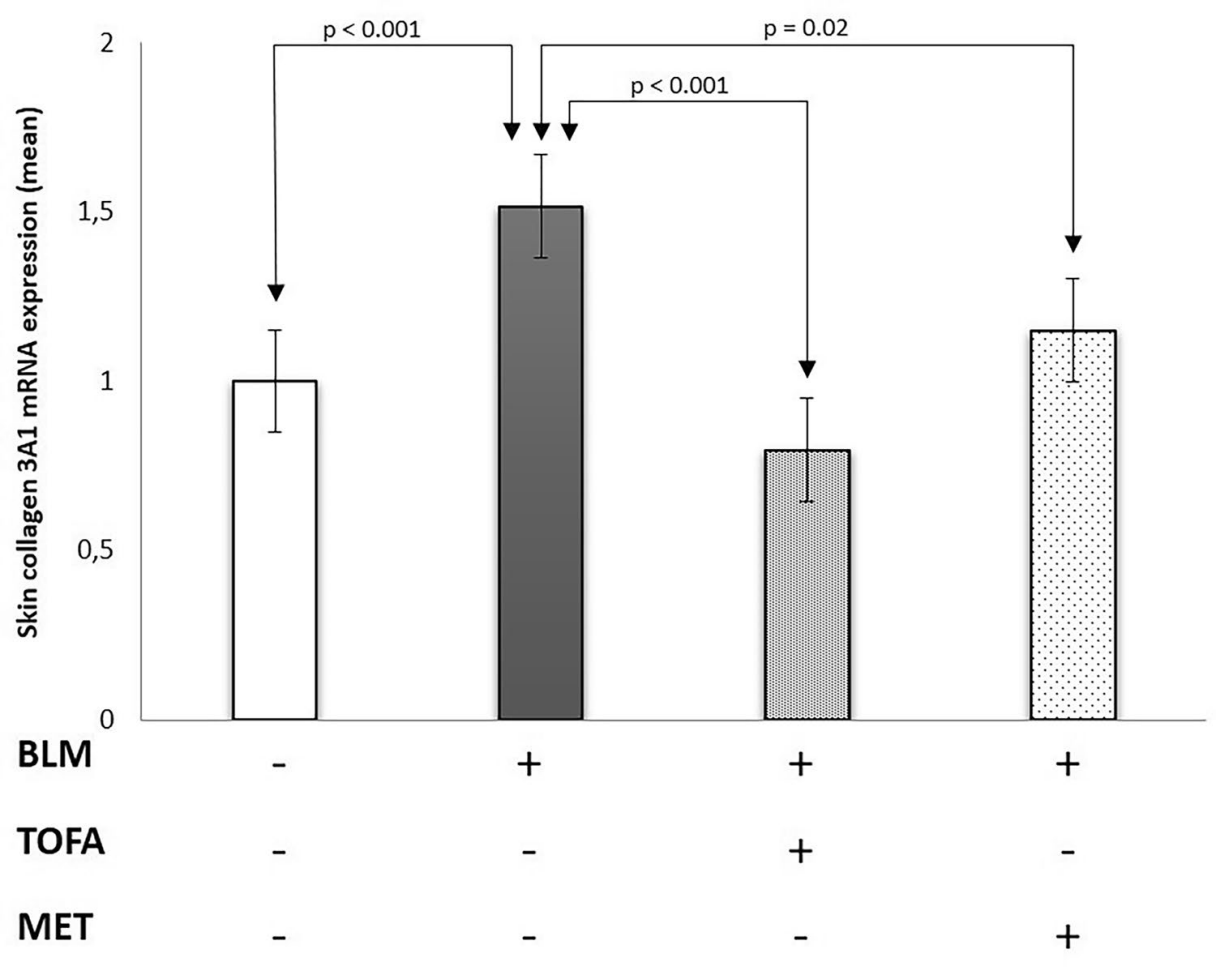
Skin collagen 3A1 messenger RNA expression in the study groups.

**Figure 4:**
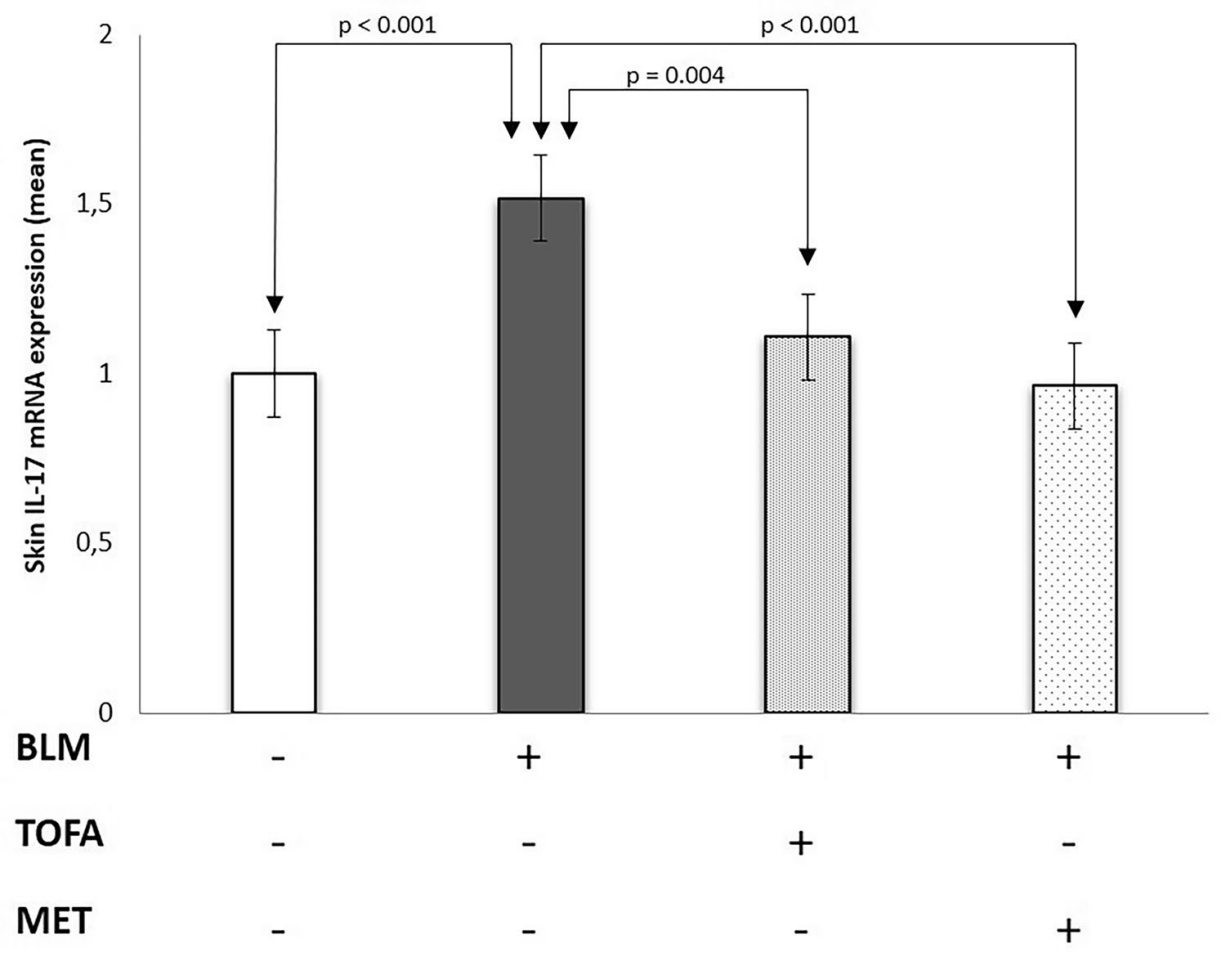
Skin interleukin (IL)-17 messenger RNA expressions in the study groups.

**Figure 5:**
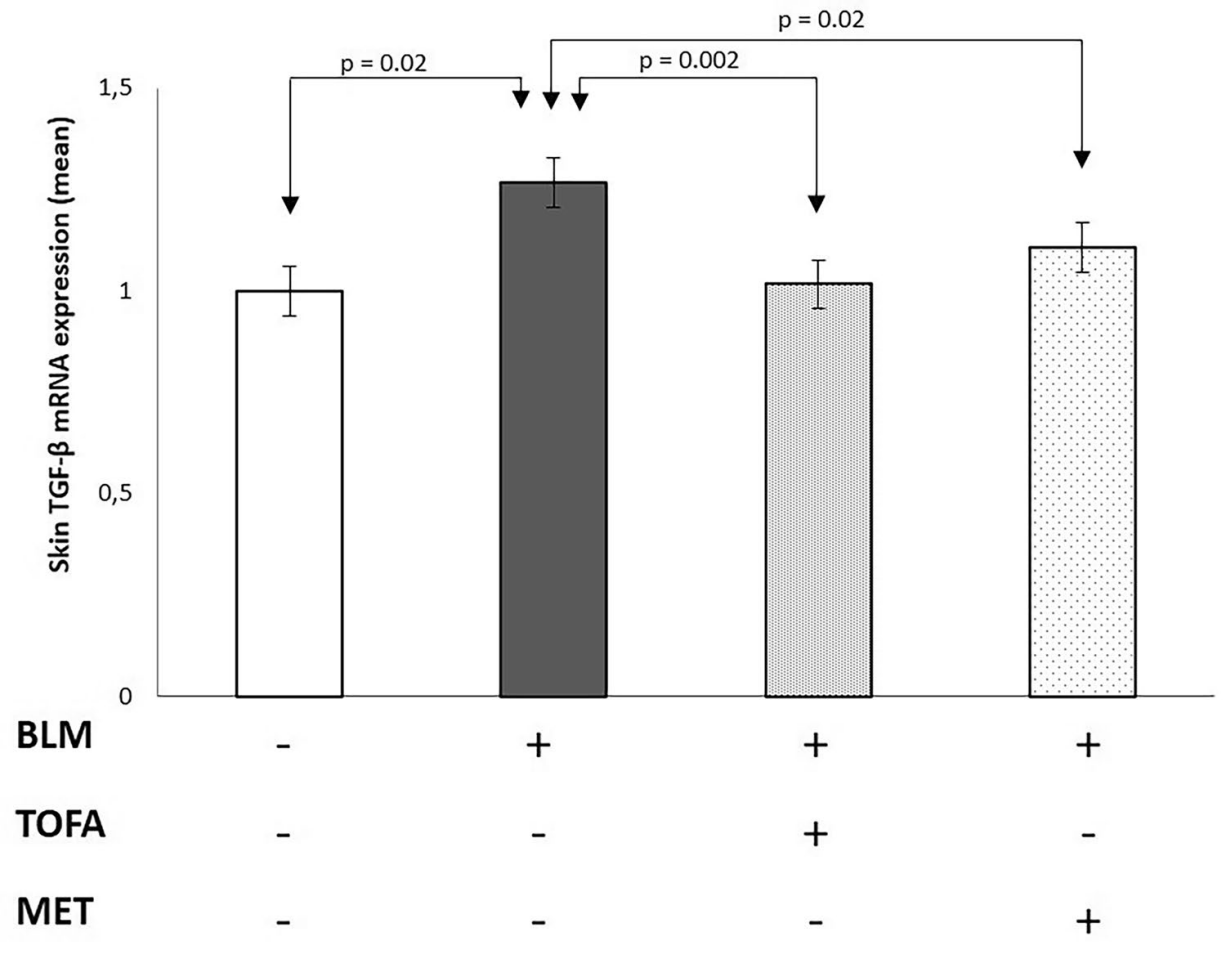
Skin transforming growth factor beta (TGF-β) messenger RNA expressions in the study groups.

When compared with the control group, the mRNA expressions of collagen 3a1, IL-17 and TGF-β were increased by 51%, 51% and 33%, respectively, in the skin of the BLM-applied sham group (p < 0.001, p < 0.001 and p = 0.02, respectively) (Table 2). In addition to, when compared with the control group, the tissue levels of collagen 3a1 and IL-17 were increased by 58% and 59%, respectively, in the skin of the BLM-applied sham group (p = 0.007 and p = 0.002, respectively) (Table 3). Skin collagen 3a1, IL-17 and TGF-β mRNA expressions were decreased by 47%, 27% and 33%, respectively, in the TOFA group when compared with the BLM-applied sham group (p < 0.001, p = 0.004 and p = 0.002, respectively). Similarly, the tissue levels of collagen 3a1 and IL-17 were decreased by 35% and 31%, respectively, in the TOFA group when compared with the BLM-applied sham group (p = 0.01 and p = 0.02, respectively). Skin collagen 3A1, IL-17 and TGF-β mRNA expressions were decreased by 19%, 40% and 25%, respectively, in the MET group when compared with the BLM group (p = 0.12, p < 0.001 and p = 0.02, respectively) (Table 2) (Figs. 3, 4 and 5). Besides, the tissue levels of collagen 3A1 and IL-17 were decreased by 21% and 39%, respectively, in the MET group when compared with the BLM-applied sham group (p = 0.14 and p = 0.001, respectively).

**Table 2:** Mean gene expression of collagen 3A1, IL-17 and TGF-β in skin tissue of study groups.

	PBS (n = 10)	BLM (n = 10)	TOFA (n = 10)	MET (n = 10)	p
Collagen 3a1	1 ± 0.6	1.5 ± 0.1*	0.8 ± 0.4^≠≠≠^	1.2 ± 0.4	0.04
IL-17	1 ± 0.2	1.5 ± 0.1**	1.1 ± 0.3^≠≠^	0.9 ± 0.2*^,≠≠^	< 0.001
TGF-β	0.9 ± 0.1	1.2 ± 0.4*	0.8 ± 0.2^≠≠^	0.9 ± 0.1^≠^	0.003

*BLM* bleomycin, *MET* metformin, *PBS *phosphate-buffered saline, *TOFA tofacitinib*.

Data were expressed as mean ± standard deviation.

When compared with the PBS group: *p < 0.05, **p < 0.001.

When compared with the BLM group: ^≠^p < 0.05, ^≠≠^p < 0.01, ^≠≠≠^p < 0.001.

**Table 3:** The levels of collagen 3a1 and IL-17 in skin tissue of study groups.

	PBS (n = 10)	BLM (n = 10)	TOFA (n = 10)	MET (n = 10)	p
Collagen 3a1 (pg/mL)	100.2 ± 31.2	158 ± 51.1*	103.6 ± 31.3^≠≠^	126.4 ± 40.2	< 0.001
IL-17 (pg/mL)	44.6 ± 15.1	70.2 ± 17.3*	48.3 ± 20.2^≠^	43.1 ± 13.7*^,≠≠^	0.003

*BLM* bleomycin, *MET* metformin, *PBS *phosphate-buffered saline, *TOFA tofacitinib*.

Data were expressed as mean ± standard deviation.

When compared with the PBS group: *p < 0.01.

When compared with the BLM group: ^≠^p < 0.05, ^≠≠^p < 0.01.

## Discussion

Although there are significant developments in the field of treatment currently, the search for an effective treatment for complex-heterogeneous SSc disease continues. The lack of effective treatment in patients with SSc whose ‘why and how’ has not been fully explained yet, leads to loss of workforce and deterioration in quality of life and negatively affects the financial burden^19^. One of the important causes of morbidity and mortality in patients with SSc is the progression toward fibrosis due to worsening of inflammation. Unfortunately, there is no effective treatment for fibrosis. As a result, fibrosis plays a vital role in the failure of the treatment of SSc. In this respect, studies concerning the treatment and pathogenesis of fibrosis in SSc are very important^20^.

TGF-β is an important profibrotic cytokine that increases the production of collagen and extracellular matrix by proliferating myofibroblasts. TGF-β stimulates fibroblasts through different pathways, such as Smad2/3, STAT3 and MAPK. In addition, fibroblast growth factor that triggers fibrosis stimulates fibroblasts through the JAK/STAT3 pathway. However, the JAK/STAT3 pathway is activated by the effect of IL-6, polarising naive T cells to Th17 cells, and as a result, the levels of IL-17 increase^7,11,21–23^. The elevated levels of IL-17 in the skin of patients with SSc can stimulate fibrosis by showing a synergistic effect with TGF-β. In another study, IL-17 was found to promote the differentiation of fibroblasts into myofibroblasts. Moreover, this effect has been shown to be mediated by the MAPK signaling pathway^24^. Millar et al. demonstrated that IL-17 signalling increased collagen type 3 levels^25^. JAK/STAT3 pathway, which is the intersection point of these pathogenic mechanisms, is thus gaining a special importance in the treatment of fibrosis.

JAK inhibitors affect the activity of T, B, macrophages and dendritic cells along with cytokine signalling^22,26^. Phosphorylated JAK1, JAK2, JAK3 and STAT3 levels were found to be increased in skin tissues of patients with SSc compared to healthy controls6. In the study conducted by Pedroza et al., the JAK/STAT3 pathway has been shown to have an important role in fibroblast-myofibroblast differentiation^27^. In another study, it has been shown that STAT3 is associated with fibroblast activation^21^. O’Reilly et al. demonstrated that STAT3 blockade reduced collagen expression in the dermal fibroblasts of systemic sclerosis patients^8^.

TOFA, a first-generation JAK inhibitor, may be a potential therapeutic agent in the treatment of SSc in terms of affecting inflammatory and fibrotic pathways^28^. Various studies have been carried out in this area related to TOFA. In the experimental scleroderma study, it has been shown that with TOFA treatment, there is a decrease in skin thickness, active STAT3 and collagen levels. However, in the same study, healing effect of TOFA on skin fibrosis could not be demonstrated when not initiated in the early period^6^. In another experimental study, the TOFA treatment was shown to improve fibrosis in skin and lung tissue by reducing T lymphocyte, CD4 + T lymphocyte levels that produce IL-13 and IL-17, B lymphocyte levels that produce IL-6, profibrotic macrophage levels and collagen synthesis^24^. Interestingly, Komai et al. reported that the modified Rodnan skin score (mRSS) rapidly improved on the 4th day with TOFA treatment in patients with SSc^29^. In their study on patients with SSc, Karaliova et al. found a 12.8% decrease in skin thickness with TOFA treatment compared to baseline at the 26th week^30^. In another study conducted with a small number of patients, it was found that the modified Rodnan skin score (mRSS) decreased more than cyclophosphamide and mycophenolate mofetil in the 1st and 6th months after TOFA treatment in patients with diffuse SSc^31^. However, no significant improvement was observed in mRSS with TOFA treatment in patients with diffuse SSc in phase I/II placebo-controlled study (NCT0327407). The low number of patients in this study may have affected the results^32^.

In our experimental study, we found a reduction in skin thickness and fibrosis with TOFA treatment. In addition, skin IL-17 and TGF-β mRNA expression, collagen level, and myofibroblast number were decreased in the TOFA group. These results support that TOFA has both anti-inflammatory and anti-fibrotic effects.

AMPK enzyme shows anti-inflammatory effects by inhibiting many signalling pathways. The activity of the AMPK enzyme is reduced in fibrotic conditions. AMPK enzyme can suppress JAK/STAT activation at both receptor and nucleus level. It inhibits pro-inflammatory gene expression by suppressing STAT-DNA binding at the nucleus level.

Metformin is one of the most commonly used cost effective antidiabetic treatment agents in the world. MET shows effects on inflammation and immune system by activating AMPK^12,33^. In addition, MET inhibits the mTOR signalling pathway through AMPK. Phosphorylated mTOR levels in fibroblasts were found to be increased in patients with SSc. In a study, it was shown that fibroblasts were suppressed with mTOR inhibition^34^. In addition, it has been shown that the mTOR signalling pathway leads to Th17 activation^35^.

It has been shown that MET therapy prevents STAT3 activation in CD4 + T cells and inhibits Th17 differentiation^36^. In addition, it has been shown that MET inhibits collagen synthesis stimulated via the SMAD pathway via TGF-β by activating the AMPK enzyme^37^. Moreover, it has been found that MET treatment reduces IL-17 levels^38^. In our study, the suppressive effect of MET treatment on IL-17 was remarkable. In a study conducted in the experimental scleroderma model, it was found that skin fibrosis was suppressed with MET treatment^39^. In our study, skin IL-17 and TGF-β mRNA expression and collagen level decreased and skin fibrosis was suppressed with MET treatment.

The limitation of our study is that the T cell subtypes were not evaluated and immunohistochemistry staining were not made.

In conclusion, TOFA and MET treatments suppressed tissue IL-17 and TGF-β levels, the productions of collagen, the infiltrations of inflammatory cells and the amount of fibrosis in an experimental BLM-induced dermal fibrosis model. These findings suggest that the therapeutic agents affecting the JAK/STAT pathway and IL-17 cytokine signal are candidate to evaluate in the treatment of SSc in the future studies.
